# A clinical medicine level test at Jinan University School of Medicine reveals the importance of training medical students in clinical history-taking

**DOI:** 10.7717/peerj.15052

**Published:** 2023-03-27

**Authors:** Xianjun Meng, Mingya Zhang, Wei Ma, Xin Cheng, Xuesong Yang

**Affiliations:** 1 School of Medicine, Jinan University, Guangdong, Guangzhou, China; 2 The First Affiliated Hospital of Jinan University, Guangdong, Guangzhou, China; 3 Division of Histology and Embryology, Key Laboratory for Regenerative Medicine of the Ministry of Education, Medical College, Jinan University, Guangzhou, Guangdong, China

**Keywords:** Clinical Medicine Level Test (CMLT), Objective Structured Clinical Examination (OSCE), Medical history-taking, Standard Patients (SP), Communication

## Abstract

**Backgrounds:**

Training in the basic interview skills of clinical history-taking has always been a significant component of medical education.

**Purpose:**

This study was designed to identify the factors influence medical students’ history-taking skills learning and develop a way to improve these skills.

**Methods:**

We firstly analysed the academic performance of medical students at Jinan University School of Medicine in different disciplines of the Clinical Medicine Level Test (CMLT), to ensure the students have obtained comprehensive medical education prior to beginning their clinical internships. Next, we conducted a survey among the CMLT participants to seek the underlying causes and corresponding measures to improve history-taking in the future. Before these medical students entered their fifth-year clinical practice, we finally provide them with pre-internship training, including the history-taking workshops with standard patients (SP).

**Results:**

The analysis of the clinical skill sections of the CMLT revealed that the students performed significantly better on clinical operations from multiple disciplines than on medical history-taking. Principal component analysis of the survey questionnaire indicated that the capability of history-taking, course assessments, and awareness of the value of medical history-taking emerged as the key factors forming a cohesive clue for sustaining history-taking implementation. The intervention workshops of employing SP had a positive impact, as evidenced by the students’ feedback and suggestions for improving their ability of history-taking.

**Conclusions:**

This study suggests that strengthening of medical history-taking training is indispensable for training qualified medical students. Workshops with SP is a successful teaching strategy for practicing history-taking, allowing students to spot minute errors and training communication skills.

## Introduction

As a certified physician, the ability to gather an accurate medical history through patient interviews is an essential skill. The process of eliciting relevant personal, psychosocial, and symptom information from a patient is defined as “history-taking”, which contributes to obtaining useful information in formulating a diagnosis and providing medical care to the patient (Keifenheim et al., 2015). Despite numerous technological advancements, such as imaging and genetic testing, that have significantly altered medical diagnosis and treatment over the past few decades, none of these scientific advances can completely replace clinical history-taking, which is considered a fundamental core competency of a physician (Novack et al., 1993). This is because collecting a patient’s medical history through interviews could significantly aid in determining the severity of a patient’s condition, identifying primary issues, and responding to patients’ emotions (Fortin et al., 2002; Hatem et al., 2007). Consequently, training in interviewing skills for obtaining a clinical history has long been a major component of medical education.

Effective communication within the medical interview contributes to the accuracy of diagnoses. Medical educators continue to emphasize the importance of teaching communication skills, as evidenced by the United States Medical Licensing Examination (USMLE) testing both the integrated clinical encounter (ICE) and communication and interpersonal skills (CIS) within the Step 2 Clinical Skills exam (CS) (Dong et al., 2015). However, it is difficult to assess communication skills through inauthentic means, such as a written test, because it requires *in vivo* demonstration. As a result, the objective structured clinical examination (OSCE), an assessment designed to measure the performance of medical tasks in a high-fidelity way, has been extensively employed to evaluate the effectiveness of medical students’ history-taking and communication skills (Jani et al., 2020). OSCE was firstly introduced in 1975 as a standardized tool for objectively assessing clinical competencies, including history-taking, physical examination, communication skills, data interpretation *etc* (Khan et al., 2013). On OSCE, medical students’ performance typically varies with rating scales and/or checklists by supervisory clinician examiners, indicating that OSCE provides a relatively fair and accurate measurement for evaluating medical students’ competency in communication, history-taking, physical examination, clinical case analysis, application of medical knowledge, and integration of fundamental clinical skills, which are required for future clinical practice (Cömert et al., 2016). Due to its advantages in comprehensively examining the clinical competencies of medical students/trainees (Ananthakrishnan, 1993; Huber, 2003), we were able to objectively evaluate medical students’ performance in a simulated work environment of OSCE, which is strictly designed to be as close to real-world scenarios as possible for future practice (Ghouri et al., 2018).

In the contemporary world, many medical schools and regulatory authorities attempt to establish accurate and reliable competency assessments to evaluate the learning outcomes of medical students, interns, and trainees. The National Medical Licensing Examination (NMLE) system has been developed in many countries to control the quality of newly licensed physicians in everyday medical practice (Han et al., 2020). In China, the NMLE has been implemented for more than two decades since the promulgation of the Law on Practicing Doctors in 1999 (Wu et al., 2014; Xin, Su & Yang, 2009; Shi, 2010). At the same time, the National Medical Examination Center (NMEC) launched the Clinical Medicine Level Test (CMLT) before medical students’ final-year internship, as a valid and reliable assessment for assessing their learning outcomes in both basic and clinical medical sciences. CMLT is administered in most medical universities, and is regarded as a pretest of the NMLE because it usually exhibits the same tendency as the NMLE, as reported by NMEC. The results of CMLT, which is administrated one to two years before NMLE at most medical schools, are regarded as a predictor and guide for subsequent undergraduate and postgraduate education, as well as residency training. Both NMLE and CMLT have the same design and contain two aspects: one is related to the cognitive component assessed by a written examination, while the other is clinical skills assessed by OSCE. Epstein stated that the objectives of the assessments in medical education are: (1) assessment of analytical and problem-solving abilities; (2) identification of incompetent physicians; and (3) selection of applicants for advanced training (Epstein, 2007). Therefore, CMLT not only identifies students early in their training whose clinical skills are developing slower than their peers, but also helps investigate the problems in current medical education that need to be addressed.

Through analyzing the medical students’ CMLT scores and collecting their feedback, this study has the following purposes: 1. Identifying the gaps between our current teaching approaches and development of medical students’ clinical abilities; 2. Establishing some post-intervention practice to cover the gaps; and 3. Improving the quality of medical students training continuously. The problem focused in this study is the deficiencies in medical history-taking training identified by CMLT score analyzing, and subsequent corrective interventions.

## Methods

### Participants and CMLT

**Figure 1:**
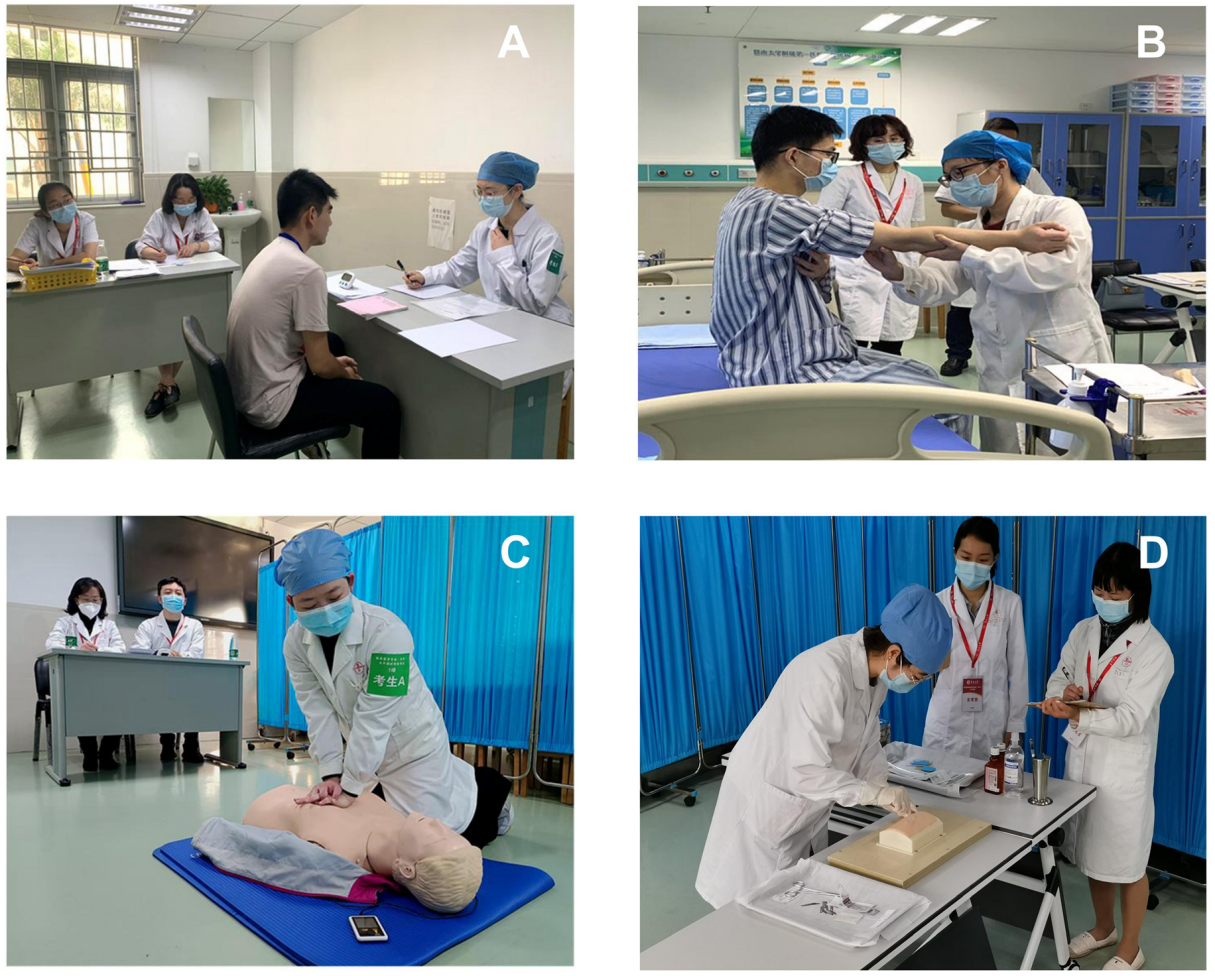
The typical scenarios shown in the various stations of CMLT. (A) Taking medical history. (B) Physical examinations. (C) Cardiopulmonary resuscitation. (D) Blood was collected by venipuncture.

In total, 168 students at Jinan University School of Medicine attended the CMLT. These participants were fourth-year undergraduates in clinical medicine who were enrolled in 2017. The examinees were 91 (54.17%) male and 77 (45.83%) female students; the average age was 22.42  ± 1.18 years old; 129 (76.79%) students were from mainland China and 39 (23.21%) were from overseas; 147 (87.50%) students studied medicine in Chinese, and 21 (12.50%) students studied medicine in English. In the spring semester of 2021, the CMLT was administered to medical students who were in the final year of their studies, prior to their internships. The examination included both written and OSCE components, with six stations covering medical history-taking, physical examinations, cardiopulmonary resuscitation, blood collection by venipuncture, and surgical suturing (Fig. 1). The results of the examination at each of the six stations were compiled by subject and categorized into four groups: internal medicine operation, emergency operation, surgical operation, and medical history-taking. The four parts were statistically described and analyzed. To enable comparison of scores, the T-score normalization method was used for OSCE scores of medical students, based on a previous report (Williams et al., 2019).

### Participants feedback

After analyzing the CMLT scores, each participant was requested to complete a questionnaire on SoJump (Ranxing, Changsha, China), which is an online survey platform in mainland China, aimed at collecting medical students’ learning experiences on history-taking before the CMLT. Additionally, the questionnaire aimed to gather the undergraduates’ feedback and attitudes towards history-taking. The survey included a total of 16 items, with three related to demographic information and 13 about medical students’ learning experiences before the CMLT. Participants were required to rate their experience using a 5-point Likert scale, with the range of “1 = strongly disagree” to “5 = strongly agree”. The last two items of the survey inquired about the medical students’ preference and training methods regarding history-taking. To ensure that the questionnaire was well-received by Chinese medical students, it was created in Chinese (see Appendix 1 for the translated English version). Prior to administration, the questionnaire was pilot-tested on ten college students who did not participate in the CMLT, Based on their feedback, and a panel discussion was organized by two senior faculty members to address the clarity of the survey and further assess its items.

Based on the survey results, we conducted history-taking workshops during the four-day pre-internship training for students’. We invited twenty students to participate in focus group interviews with the first two authors after the training. The interview topics primarily revolved around three questions: (1) What do you believe is the most important aspect of medical history-taking? (2) What do you consider the most effective way to master the skills of medical history-taking? (3) What kinds of teaching methods are more helpful to your studies? Participation in the survey and interviews was voluntary, and all respondents were informed of the study’s purpose and procedures. They were provided with written consent forms and informed that they could withdraw at any time. To maintain confidentiality, the materials were de-identified in the transcripts, and the data were only accessible to the authors. This study was conducted in accordance with the Declaration of Helsinki and was approved by the Ethics Committee of Jinan University (No. JNUKY-2021-039).

### Statistical analysis

SPSS 22.0 software was used to conduct the statistical analyses. Descriptive statistics was performed to assess the demographic characteristics of all participants involved in the study. The independent samples *t* test (2-tailed) was used to compare (1) the average OSCE scores of four different subjects and the average 6-station OSCE score of all the participants; and (2) the average score of individual test contained in each OSCE subject and the average score of each OSCE subject. Paired samples *t* test was used to compare the average scores for evaluating the medical students’ history-taking and clinical operation skills of internal medicine in OSCE. The data obtained from the questionnaires were analyzed using Cronbach’s alpha test to determine the internal consistency of the responses. Factor analysis (principal component analysis) was employed as a useful tool for characterizing and quantifying the influencing factors of the students’ history-taking skills (Chesser et al., 2004). The rotation method was Varimax. Number of factors were determined by the eigenvalues extracted greater than 1. The results of the statistical analyses are presented as the mean ± standard deviation (SD) and were considered statistically significant when *P* < 0.05.

## Results

### The analysis of CMLT scores revealed that medical students’ inability to take a patient’s medical history was their greatest weakness

We first implemented the standard fractional transformation of the students’ scores, because the OSCE scores were not normally distributed. The students’ original scores at each station were converted into standard scores using the following formula: T = 10Z + 50 (T stands for fraction of T; Z stands for standard fraction). As a result, the average standard score of the 168 OSCE participants is 50. Four subjects classified by discipline were statistically examined (Table 1). The descriptive statistical results indicate that the average scores of both internal medicine operation and medical history-taking were lower than the other average scores, particularly the score of medical history-taking was significantly lower than the average OSCE score. This outcome suggests that there must be some inadequacies in current clinical history-taking education.

**Table 1:** A summary of participants’ scores on different OSCE subjects.

**Subjects**	***n***	**Mean (SD)**	**Median**	**Skewness**	**Peakness**	***t***	***P***
Internal medicine operation	168	48.66 (10.16)	51.53	−1.71	3.91	−1.71	0.090
Emergency operation	168	54.85 (6.69)	56.07	−2.85	16.18	7.15	** <0.001**
Surgical operation	125	54.56 (7.13)	55.16	−2.91	16.30	9.39	** <0.001**
Medical history-taking	168	45.43 (6.84)	46.99	−0.76	0.15	−8.65	** <0.001**

**Notes.**

Independent *t*-tests were employed to compare the average OSCE scores of four different subjects with the average 6-station OSCE score of all the participants, which was normalized to 50.

n The number of participants who selected the tests of the subjects SD standard deviation

The values in bold show the statistically significant difference.

To further validate the preliminary analysis of the results mentioned above, we compared the average score of each test from the 168 participants with the overall average scores of each subject, including history-taking, surgical operation and emergency operation. The results showed that the most significant difference occurred in the items related to history-taking, whereas there were a few significant differences in either surgical or emergency operations (Table 2).

**Table 2:** Comparison of each test’s average score to each OSCE subject.

**Subjects**	**Mean score of the subject (SD)**	**Number of contained tests**	***n***	**Mean score of the test (SD)**	***t***	***P***
Internal medicine operation (*n*= 168)	48.66 (10.16)	1	55	46.45 (6.17)	−2.66	**0.010**
				2	56	44.67 (7.81)	−3.82	** <0.001**
				3	57	45.21 (6.43)	−4.05	** <0.001**
				4	41	48.83 (8.98)	0.12	0.906
				5	43	50.20 (10.93)	−0.63	0.534
				6	42	48.03 (9.99)	0.91	0.367
		7	42	46.45 (6.17)	−0.41	0.683
Surgical operation (*n*= 125)	54.56 (7.13)	1	22	55.62 (5.71)	0.87	0.395
				2	21	52.78 (6.28)	−1.30	0.210
				3	20	58.07 (3.77)	4.16	**0.001**
				4	21	54.21 (6.26)	−0.26	0.801
				5	20	56.03 (4.72)	1.39	0.181
		6	21	50.84 (11.58)	−1.47	0.156
Emergency operation (*n*= 168)	54.85 (6.69)	1	168	57.23 (4.93)	−0.62	0.541
		2	22	54.19 (4.96)	6.27	** <0.001**

**Notes.**

Independent *t*-tests were employed to compare the average score of each test with that of each OSCE subject. The internal medicine operation consists of 7 tests, from which the examinee may select 2 to be tested. The surgical operation includes 6 tests, one of which may be selected for testing. The emergency operation consists of 2 tests, the 1st of which is required and the 2nd of which can be selected at random.

SD standard deviation *n* The numbers of the participants who responded the tests

The values in bold show the statistically significant difference.

We then proceeded to compare the OSCE scores between history-taking and general hospital clinical operation of internal medicine. The results indicated that the average score for history-taking was significantly lower than that of general hospital clinical operation (Table 3).

**Table 3:** Comparison of the average scores for evaluating the medical students’ history- taking and clinical operation skills of internal medicine.

**Subjects**	***n***	**Mean (SD)**	***t***	***P***
Medical history-taking	168	45.44 (6.84)	12.26	** <0.001**
Clinical operation skills	168	52.28 (6.81)		

**Notes.**

Paired samples *t*-tests was employed to compare the average score for evaluating the medical students’ history-taking and clinical operation skills of internal medicine in OSCE.

*n* The numbers of the participants who participated the examination SD standard deviation

The values in bold show the statistically significant difference.

### The investigation for the primary causes of low history-taking scores among the students

To explore the key factors influencing students’ performance in medical history-taking, we administered a survey questionnaire. All the 168 CMLT participants completed the questionnaire. The Cronbach’s *α* of the 11 items on the participants’ acknowledgement and assessments about medical history-taking was 0.680, indicating a good reliability of the survey tool (Appendix 1). The principal factor analysis of the questionnaire showed that three key factors accounted for 52.26% of the total variance, including the ability for history-taking, comments on the course, and awareness of the value of history-taking. In particular, the scores were extremely low when students answered the item “I am satisfied with the current learning strategies of medical history-taking” (Table 4). Other two items of the survey evaluated the medical students’ preference regarding the learning strategies of history-taking. The results showed that instructor lectures were the primary method of instruction currently; however, students requested that more interviews with actual patients and standard patient (SP) training modules would be incorporated into future teaching approaches (Table 5).

**Table 4:** Principal factor analysis of the questionnaire on participants’ feedback.

**Items**	**Mean (SD)**	**Ability to obtain a medical history**	**Comments on the course**	**Awarenessof the value of medical history-taking**
10. I have good communication skills in the process of medical history-taking.	3.50 ± 0.69	0.763		
9.I have a solid understanding of interrogating skills in the context of obtaining a medical history.	3.45 ± 0.82	0.709		
4.I am familiar with the fundamental steps of the medical history taking process.	3.82 ± 0.72	0.651		
8.In the process of obtaining a medical history, I have a firm handle on my ability to think inquiringly.	3.54 ± 0.75	0.601		
11.I have a solid understanding of the humanistic qualities and skills required for taking a medical history.	3.24 ± 0.59	0.554		
6. I believe systematic training can enhance the skill of taking a patient’s medical history.	4.47 ± 0.86		0.703	
14. I am satisfied with the teachers who teach medical history taking.	3.89 ± 0.71		0.605	
13.I think the class hour arrangement of medical history taking is reasonable.	3.90 ± 0.69		0.595	
12. I am satisfied with the current learning method for medical history taking.	2.81 ± 0.61		−0.450	
5.I believe that obtaining a patient’s medical history is essential to clinical reasoning and decision-making in diagnosis and therapy.	4.31 ± 0.65			0.725
7. I am interested in learning medical history-taking.	3.60 ± 0.72			0.580
Cumulative variance %	52.26	24.91	14.63	12.72

**Notes.**

Extraction methods: Principal component analysis was used for factor extraction. The rotation methods was Varimax Kaiser normalization (KMO = 0.685). Rotation converged in 9 iterations. *n* = 109. Cronbach’s *α* = 0.680.

SD standard deviation

**Table 5:** The results of surveying medical history-taking and learning approaches.

**Items**	Roleplay ***n*(%)**	Case-based learning ***n*(%)**	**SP training** ***n*** **(%)**	**Interview with real patients** ***n*** **(%)**	Instructor teaching ***n*(%)**
What was the most important way for you to learn medical history-taking during the internship?	7 (6.42)	9 (8.26)	12 (11.01)	25 (22.94)	56 (51.38)
What do you think is the best way to learn medical history-taking?	11 (10.09)	6 (5.50)	28 (25.69)	31 (28.44)	33 (30.28)

**Notes.**

SP standard patient *n* The numbers of the students who selected the response anchors

### The intervention measures for improving history-taking ability had a positive impact

#### Workshops

Based on the deficiencies in history-taking revealed by the CMLT, we implemented a series of remedial measures, which included organizing workshops on history-taking during a 4-day pre-internship training program, as a specific form of improvement. In the targeted training, we took into consideration the students’ learning preference, and employed SP to introduce the procedure of history-taking and gave the students deliberate practice. The SP interviewing scenarios were recorded and reviewed with feedback and discussion session facilitated by either group members or the teacher. Throughout the review process, some subtle issues in the beginning of the interview, problem-solving, communication, and summary were perceived, until a more standardized structure for history-taking was established. The SP interviews in the workshop were supplemented by mini-lectures, small group exercises such as role-play and self-reflection.

#### Students feedback

After the pre-internship training, 20 students were interviewed to assess the effectiveness of these training modules. Table 6 shows their representative opinions and suggestions. The students believed that understanding the process of history-taking and knowing how to efficiently use history-taking in practice were very important. Additionally, they felt that accumulating medical knowledge and increasing learning interests were equally important. The students also indicated that problem-based learning (PBL) was a preferred approach for improving their ability to efficiently perform history-taking in practice. These comments suggest that the remedial measures for history-taking have contributed to the improvement of students’ history-taking skills.

**Table 6:** Representative comments from the interviews with the pre-internship training students.

**Items**	**Representative comments**
1. What do you believe is the most important aspect of medical history-taking?	-Clear consultation process
	-Proficiency in medical history-taking
	-Application of professional knowledge
2. What, in your viewpoint, is the most effective way to master the skills of medical history-taking?	-Mastery of medical knowledge
	-Improvement of learning interest
	-Improvement of enthusiasm and subjective initiative
	-Ability to clinical thinking
	-Sense of teamwork
	-Effective teaching method
3. What kinds of teaching methods are more helpful to your studies?	-*Problem-based learning (PBL)*. During PBL, the problems serve as the learning objectives. We can increase our understanding and mastery of theoretical knowledge by thinking about problems, expressing our views, and consulting professional knowledge and reference materials in the process.
	-*Simulated SP teaching*. Teachers simulate the pathophysiological characteristics of clinical patients, allowing us to diagnose and treat them based on their clinical symptoms. This method can overcome the difficulty of finding targeted cases by designing medical records, actually simulating clinical situations, and modifying roles based on needs. Teachers can also act as evaluators to assess our performance.

## Discussion

Prior to internships in China, the CMLT evaluates the effectiveness of teaching and the learning outcomes of medical students. Although not yet mandatory for all Chinese medical students, accumulated experience in recent years has shown that it is beneficial in increasing the practitioners’ NMLE passing rate (Lio et al., 2016; Zhang et al., 2019).

This study initially compared the CMLT performance of several groups of medical student cohorts at Jinan University School of Medicine in 2021. The primary conclusion of the analysis was that clinical history-taking had poor performance, indicating that there may be inherent or external factors affecting the learning outcomes of medical students. Subsequently, we focused on the causes of medical students’ unsatisfactory performance in taking patients histories. We conducted an analysis of the specific variation to provide useful information for enhancing the effectiveness of history-taking instruction.

In recent decades, medical history-taking has been prioritized in clinical medical education, with numerous medical schools developing consensus guidelines emphasizing its significance (Novack et al., 1993). Clinical history-taking is considered an efficient way of obtaining more complete and accurate patient information to guide disease diagnosis and treatment (Keifenheim et al., 2015; Schechter et al., 1996), and interpersonal ability is also considered a core skell of a qualified physician (Hargie et al., 1998; Novack et al., 1993). Retired physician David Levine said that listening to patients is not enough, as history taking is a complex skill that requires skilled clinical reasoning to choose the right questions and interpret the answers to decide what to ask or do next (Levine, 2017). Deliberate practice from seeing more cases is an effective way to improve skills, but formal training is necessary for inexperienced medical students. In this study, we found that our medical students’ performance was generally unsatisfactory in history-taking, especially when compared to emergency cardiopulmonory resuscitation (CPR), and surgical procedures such as suturing, fixation, and bandaging (Tables 1–3). The principal factor analysis of the questionnaire from the participating students revealed that the students recognized the importance of history-taking skills, and had some evaluations and suggestions for effective history-taking training (Table 4). This result indicated that the students were eager to develop their history-taking skills.

On the other hand, the students were dissatisfied with the current teaching approaches for medical history-taking, and they wished for more opportunities to contact actual patients or be trained with SPs that could mimic clinical scenarios while reducing the time for classroom instruction on history-taking (Table 5). This attitude echoes a previous report in which training students in history-taking skills with televised demonstrations and SPs was more efficient than traditional formats (Maguire, Clarke & Jolley, 1977). Furthermore, this simulation training would greatly encourage medical students to shift from a traditional “questioning style” to a “functional model”, educating patients and influencing their attitudes towards the diseases they suffer from, rather than simply collecting patients’ physical condition (Keifenheim et al., 2015). Skills of communication, clinical reasoning and summary are used during medical history-taking. These non-technical skills are elements of high-level cognition, especially some of while are related to medical humanity education, which is hard to define and unify because humanistic education requires inter-professionals and its methods can vary (Spence et al., 2020). Therefore, educator are exploring the most effective methods and the optimal timing of history-taking training.

Regarding the improvement of medical students’ history-taking skills, Keifenheim et al. suggested several educational interventions. These include implementing small group workshops that involve role-playing and interviews with real patients, discussing the most common and best cases among the participants, and promoting instructive experiences and lessons through videotape reviews *etc* (Keifenheim et al., 2015). In light of our survey results, we incorporated history-taking workshops with SP into pre-internship training for medical students before they started their medical internships in the last academic year. Feedback from participating students revealed that the workshops were effective in helping them understand the standard process of history-taking and competently applying it in practice. The workshops also broadened their medical knowledge and promoted learning enthusiasm (Table 6). This finding confirmed the notion that the history-taking skills required by qualified physicians are not innate and must be particularly and repeatedly practiced throughout medical education (Kraan et al., 1990; Pfeiffer et al., 1998). The real examination of this training’s outcome would be completed by means of evaluating students’ ability to obtain an accurate medical history from patients in clinical practice. This is also our ultimate objective for the education of training medical students at medical schools.

This study has several practice implications:

1. Communication skills for medical interviews should be emphasized through effective training methods.

2. SP training for practicing history-taking can be incorporated into the routine clinical training curriculum.

3. Simulative learning environments that can replicate every step of history-taking and allow students to spot minute errors are a successful teaching strategy.

## Limitations of this Study

The present study has both strengths and limitations. The first limitation is that the conclusions were derived from one CMLT and the subsequent survey at Jinan University School of Medicine, so they may not be completely applicable to all teaching hospitals. There are numerous different teaching facilities and instructional strategies that could influence the perceptions of medical students regarding the advantages and disadvantages of medical education. Second, while we intended for the CMLT to reflect the educational situation of basic and clinical medicine and provide a beneficial supplement for the formal examination of doctors’ qualifications, there may be flaws in the structure, design and practical experience due to the limited time and scope of implementation.

##  Supplemental Information

10.7717/peerj.15052/supp-1 Supplemental Information 1 Questionnaire in English

10.7717/peerj.15052/supp-2 Supplemental Information 2 Questionnaire in Chinese

10.7717/peerj.15052/supp-3 Data S1 Raw data

## Additional Information and Declarations

### Data Availability

The following information was supplied regarding data availability: The raw data is available in the Supplemental Files.
