# Supplementary material for: A clinical medicine level test at Jinan University School of Medicine reveals the importance of training medical students in clinical history-taking
Source: PeerJ. 2023 Mar 27;11:e15052. doi: 10.7717/peerj.15052 (PMC10062337; doi:10.7717/peerj.15052)
Supplement: Supplemental Information 1 [file peerj-11-15052-s001.pdf]

The questionnaire of medical students' perception towards taking medical history

We appreciate your participation in this survey to provide feedback on the medical history section of the CMLT. The survey findings will help us identify existing issues and enhance the design and quality of the CMLT medical history section accordingly. The questionnaire consists of 16 questions, and completion will take a few minutes. There is no correct or incorrect response to these questions. Please respond based on your experience taking the medical history section of the CMLT in the fall of 2021. We guarantee that all data will remain anonymous, and no personal information will be disclosed. Again, many thanks for your participation.

The general information:

1. Your gender is _____ [Single choice]
   1) Male
   2) Female

2. How old are you? [Fill-in-the-blank question]
   I am _____ years old.

3. Which major are you from? [Single choice]
   1) Mandarin course in clinical medicine (Internship site – University Hospital)
   2) Mandarin course in clinical medicine (Internship site – 2nd Provincial Hospital)
   3) Mandarin course in clinical medicine (Internship site – Shenzhen People's

Hospital)

4) Mandarin course in clinical medicine (Overseas students; Internship site – University Hospital)
5) English course in clinical medicine (Internship site – University Hospital)

Please respond to the following questions based on your perspective and the present status of your research on medical history taking.

4. I am familiar with the fundamental steps of the medical history taking process. [Single choice]

1) Strongly disagree
2) Disagree
3) Neutral
4) Agree
5) Strongly agree

5. I believe that obtaining a patient's medical history is essential to clinical reasoning and decision-making in diagnosis and therapy. [Single choice]

1) Strongly disagree
2) Disagree
3) Neutral
4) Agree
5) Strongly agree

6. I believe systematic training can enhance the skill of taking a patient's medical history. [Single choice]

1) Strongly disagree

2) Disagree

3) Neutral

4) Agree

5) Strongly agree

7. I am interested in learning medical history taking. [Single choice]

1) Strongly disagree

2) Disagree

3) Neutral

4) Agree

5) Strongly agree

8. In the process of obtaining a medical history, I have a firm handle on my ability to think inquiringly. [Single choice]

1) Strongly disagree

2) Disagree

3) Neutral

4) Agree

5) Strongly agree

9. I have a solid understanding of interrogating skills in the context of obtaining a medical history. [Single choice]

1) Strongly disagree

2) Disagree

3) Neutral

4) Agree

5) Strongly agree

10. I have good communication skills in the process of medical history taking. [Single choice]

1) Strongly disagree

2) Disagree

3) Neutral

4) Agree

5) Strongly agree

11. I have a solid understanding of the humanistic qualities and skills required for taking a medical history. [Single choice]

1) Strongly disagree

2) Disagree

3) Neutral

4) Agree

5) Strongly agree

12. I am satisfied with the current learning method for medical history taking. [Single choice]

1) Strongly disagree

2) Disagree

3) Neutral

4) Agree

5) Strongly agree

13. I think the class hour arrangement of medical history taking is reasonable. [Single choice]

1) Strongly disagree

2) Disagree

3) Neutral

4) Agree

5) Strongly agree

14. I am satisfied with the teachers who teach medical history taking. [Single choice]

1) Strongly disagree

2) Disagree

3) Neutral

4) Agree

5) Strongly agree

15. What are the main ways of learning medical history-taking during your internship?

1) Role play

2) Case based learning

3) SP training

4) interview with real patients

5) Instructor teaching

16. What do you think is the best way to learn medical history-taking?

1) Role play

2) Case based learning

3) SP training

4) interview with real patients

5) Instructor teaching
