# Supplementary material for: A clinical medicine level test at Jinan University School of Medicine reveals the importance of training medical students in clinical history-taking
Source: PeerJ. 2023 Mar 27;11:e15052. doi: 10.7717/peerj.15052 (PMC10062337; doi:10.7717/peerj.15052)
Supplement: Supplemental Information 2 [file peerj-11-15052-s002.docx]

**病史采集学习现状调查问卷**

       您好！病史采集是临床诊治疾病的重要环节，是加强医患沟通交流、建立良好医患关系的重要手段，正确的问诊技巧和良好的沟通技能是获得系统准确病史资料的前提。为了解我院学生病史采集学习现状，进一步对病史采集教学方式方法改进，提升学生临床综合技能，请您在百忙之中填写这份调查问卷。谢谢您对我们工作的支持和对医学教学提升中的贡献！

一、基本信息
1. 您的性别 [单选题] *

| ○男 | ○女 |
| --- | --- |

2. 您的年龄：_______岁 [填空题] *

3.您是属于哪个班？ [单选题] *

| ○临床医学中文班（校本部） |
| --- |
| ○临床医学中文班（省二医） |
| ○临床医学中文班（深圳） |
| ○临床医学中文班（外招） |
| ○临床医学全英班 |

二、病史采集学习认识
4. 我对病史采集技能很熟练 [单选题] *

| ○完全不同意 | ○不同意 | ○一般 | ○同意 | ○完全同意 |
| --- | --- | --- | --- | --- |

5. 我认为病史采集对了解患者病情的很重要 [单选题] *

| ○完全不同意 | ○不同意 | ○一般 | ○同意 | ○完全同意 |
| --- | --- | --- | --- | --- |

6. 我认为病史采集学习可以通过系统训练提高 [单选题] *

| ○完全不同意 | ○不同意 | ○一般 | ○同意 | ○完全同意 |
| --- | --- | --- | --- | --- |

7. 我对病史采集很感兴趣 [单选题] *

| ○完全不同意 | ○不同意 | ○一般 | ○同意 | ○完全同意 |
| --- | --- | --- | --- | --- |

8. 病史采集过程中，我掌握了较强问诊技能 [单选题] *

| ○完全不同意 | ○不同意 | ○一般 | ○同意 | ○完全同意 |
| --- | --- | --- | --- | --- |

9. 我认为掌握病史采集技能很重要 [单选题] *

| ○完全不同意 | ○不同意 | ○一般 | ○同意 | ○完全同意 |
| --- | --- | --- | --- | --- |

10. 病史采集过程中，我的沟通能力很好 [单选题] *

| ○完全不同意 | ○不同意 | ○一般 | ○同意 | ○完全同意 |
| --- | --- | --- | --- | --- |

11. 我认为沟通技能对病史采集很重要 [单选题] *

| ○完全不同意 | ○不同意 | ○一般 | ○同意 | ○完全同意 |
| --- | --- | --- | --- | --- |

12. 我对现在病史采集学习方式很满意[单选题] *

| ○完全不同意 | ○不同意 | ○一般 | ○同意 | ○完全同意 |
| --- | --- | --- | --- | --- |

13. 现在病史采集学习课时很合理 [单选题] *

| ○完全不同意 | ○不同意 | ○一般 | ○同意 | ○完全同意 |
| --- | --- | --- | --- | --- |

14.我很满意病史采集的老师[单选题] *

| ○完全不同意 | ○不同意 | ○一般 | ○同意 | ○完全同意 |
| --- | --- | --- | --- | --- |

15. 在实习时主要学习病史采集的方式有哪些？ [单选题] *

| ○角色扮演 | ○案例分析 | ○SP训练 | ○教师讲授 | ○临床学习 |
| --- | --- | --- | --- | --- |

16. 你认为哪种方式学习病史采集最好？ [单选题] *

| ○角色扮演 | ○案例分析 | ○SP训练 | ○教师讲授 | ○临床学习 |
| --- | --- | --- | --- | --- |
